# Comparative Genomic Insights into Secondary Metabolism Biosynthetic Gene Cluster Distributions of Marine *Streptomyces*

**DOI:** 10.3390/md17090498

**Published:** 2019-08-26

**Authors:** Lin Xu, Kai-Xiong Ye, Wen-Hua Dai, Cong Sun, Lian-Hua Xu, Bing-Nan Han

**Affiliations:** 1 Lab of Marine Functional Molecules, Zhejiang Sci-Tech University, Hangzhou 310018, China; 2 College of Life Sciences and Medicine, Zhejiang Sci-Tech Univeristy, Hangzhou 310018, China; 3 Key Laboratory of Marine Ecosystem and Biogeochemistry, State Oceanic Administration & Second Institute of Oceanography, Ministry of Natural Resources, Hangzhou 310012, China

**Keywords:** *Streptomyces*, comparative genomics, secondary metabolites, biosynthetic gene clusters, phylotype, ecotype

## Abstract

Bacterial secondary metabolites have huge application potential in multiple industries. Biosynthesis of bacterial secondary metabolites are commonly encoded in a set of genes that are organized in the secondary metabolism biosynthetic gene clusters (SMBGCs). The development of genome sequencing technology facilitates mining bacterial SMBGCs. Marine *Streptomyces* is a valuable resource of bacterial secondary metabolites. In this study, 87 marine *Streptomyces* genomes were obtained and carried out into comparative genomic analysis, which revealed their high genetic diversity due to pan-genomes owning 123,302 orthologous clusters. Phylogenomic analysis indicated that the majority of Marine *Streptomyces* were classified into three clades named Clade I, II, and III, containing 23, 38, and 22 strains, respectively. Genomic annotations revealed that SMBGCs in the genomes of marine *Streptomyces* ranged from 16 to 84. Statistical analysis pointed out that phylotypes and ecotypes were both associated with SMBGCs distribution patterns. The Clade I and marine sediment-derived *Streptomyces* harbored more specific SMBGCs, which consisted of several common ones; whereas the Clade II and marine invertebrate-derived *Streptomyces* have more SMBGCs, acting as more plentiful resources for mining secondary metabolites. This study is beneficial for broadening our knowledge about SMBGC distribution patterns in marine *Streptomyces* and developing their secondary metabolites in the future.

## 1. Introduction

Bacterial secondary metabolites are defined as organic compounds that are not directly involved in the normal growth and proliferation of bacteria [1], and can be classified into several categories, such as alkaloids, antibiotics, carotenoids, pigments, and toxins [2]. Bacterial secondary metabolites play an important role in defending against adversities and increasing the survival of themselves, even their hosts, due to their antibacterial, antifungal, antitumor, and antiviral activities [3,4], meaning those organic compounds have considerable application potential in human and veterinary medicine as well as agriculture [5]. Since the initial discovery of bacterial secondary metabolites in the 1920s, they have shown a profound impact on human society [6]. Currently, marine-derived bacterial secondary metabolites with a broad range of complex structures are increasingly becoming sources of novel natural products for discovering and developing new drugs [7,8,9,10].

Genes involved in the biosynthesis of bacterial secondary metabolites are commonly organized in the secondary metabolism biosynthetic gene clusters (SMBGCs) [1,9]. The development of genomic sequencing technology facilitates the mining of marine bacterial SMBGCs [11,12,13]. Apart from core biosynthetic enzyme-encoding genes, SMBGCs generally also harbor genes encoding enzymes to synthesize specialized monomers, transporters, and regulatory elements as well as mediating host resistance [14]. Non-ribosomal peptide synthase (NRPS) and polyketide synthase (PKs) gene clusters are two main pathways for biosynthesizing bacterial secondary metabolites [15]. Those two core enzymes independently fold protein domains, operate in constructing polymeric chains, and tailor their functionalities [15]. In addition, another well-known class of SMBGCs is terpenes, which are derived biosynthetically from units of isopentenyl pyrophosphate through mevalonic acid pathway or 2-*C*-methyl-d-erythritol 4-phosphate pathway [16,17]. Because bacterial secondary metabolites improves fitness advantages of bacteria as well as their hosts and the frequency of horizontal gene transfer is high, some studies indicated that SMBGC distributions are related to the environment where bacteria live, called ecotype [18,19]. Meanwhile, recent studies demonstrated that bacterial secondary metabolite production is species-specific, which concerns phylogeny, called phylotype [20,21]. Therefore, what distribution patterns of SMBGCs are is still an open scientific question that is associated with phylotypes and ecotypes. The exploration of this question is beneficial for developing bacterial secondary metabolites.

The genus *Streptomyces* belongs to the family *Streptomycetaceae*, the order *Actinomycetales*, the class *Actinobacteria,* and the phylum *Actinobacteria* [22], and it is one of the largest group in this phylum with more than 600 species at the time of writing (http://www.bacterio.net/streptomyces.html, [23]). The genus *Streptomyces* is well known for an important source of secondary metabolites, and the portion of recently novel antibiotics discovered from this genus can reach at about 20–30% [24,25]. Further, the genus *Streptomyces* inhabits a wide range of marine habitats, including seawater [26,27], marine sediments [28,29], alga [30,31], mangroves [32,33], sponges [34,35], corals [36,37], tunicates [38,39], mollusks [40,41], etc., resulting in the fact that this genus attracts continuous attentions of researchers to find valuable secondary metabolites. Furthermore, the genus *Streptomyces* is one of the earliest genome-sequenced prokaryotes, with the genome of *S*. *coelicolor* A3(2) sequenced and reported by Bentley et al. in 2002 [42]. Hundreds of *Streptomyces* genomes have been sequenced and deposited into public databases in the recent years [43,44], leading to the increases of comparative genomic studies about this genus. While comparative genomic studies of marine *Streptomyces* are mostly related to exploring their SMBGC resources as well as diversities [45,46,47,48,49] and investigating their marine adaptation mechanisms [50,51], there is still a lack of comprehensive study concerning SMBGC distribution patterns in marine *Streptomyces*. In this study, we proposed the hypothesis that both of phylotype- and ecotype-associated SMBGCs were in the genomes of marine *Streptomyces* and performed comparative genomic methods to test this hypothesis and analyze their distribution patterns. This study is beneficial for broadening our knowledge about SMBGC distribution patterns in marine *Streptomyces* and developing their secondary metabolites in the future.

## 2. Results and Discussion

### 2.1. Genomic Characteristics and Annotation Results of Marine Streptomyces

Eighty-seven marine *Streptomyces* genomes were screened into genomic analysis by confirming their high genomic qualities with the completeness >95% and the contamination <5% (Table S1). Those strains were isolated from various sources, including seawater (*n* = 7), marine sediments (*n* = 38), cyanobacteria (*n* = 1), algae (*n* = 1), mangroves (*n* = 8), sponges (*n* = 22), corals (*n* = 3), tunicates (*n* = 2), and mollusks (*n* = 5).

**Figure 1:**
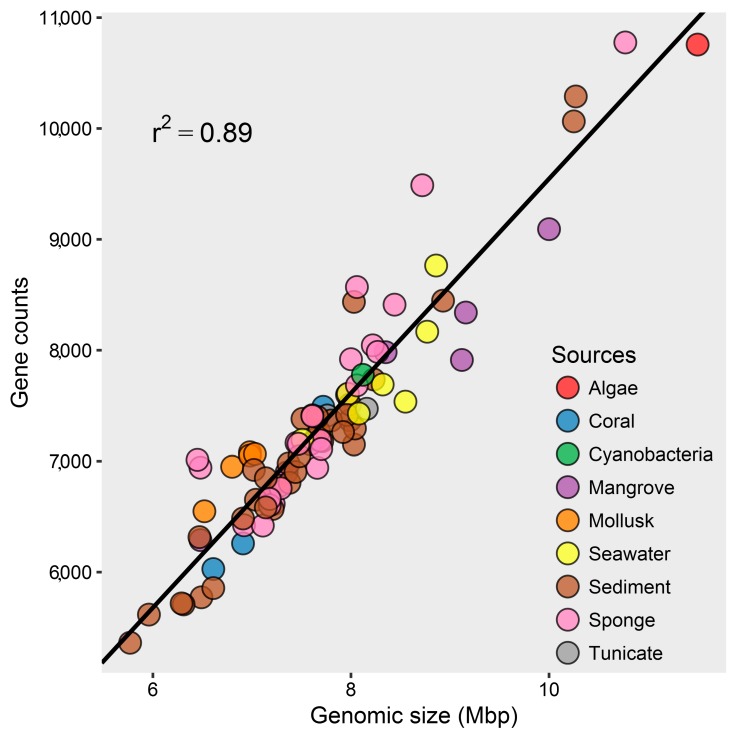
Genomic size and gene counts of *Streptomyces* derived from various marine environments.

The G+C contents of marine *Streptomyces* were 69.9–73.8 mol% (Table S1), which was in accordance with high G+C content as a typical characteristic of the phylum *Actinobacteria* [52]. Genomic sizes and gene counts of those marine *Streptomyces* genomes varied remarkably, ranging from 5.77 to 11.50 Mbp and from 5363 to 10,776 (Figure 1 and Table S2), respectively. Furthermore, the number of genes was positively correlated with the genomic size of the marine *Streptomyces* (*y* = 966.8*x* − 121.4, *r*^2^ = 0.89). Furthermore, it was found that 3978–8065 (71.15–78.9%) and 2005–3192 (27.6–38.0%) genes were assigned to Clusters of Orthologous Groups (COG) and Kyoto Encyclopedia of Genes and Genomes (KEGG) databases, respectively.

**Figure 2:**
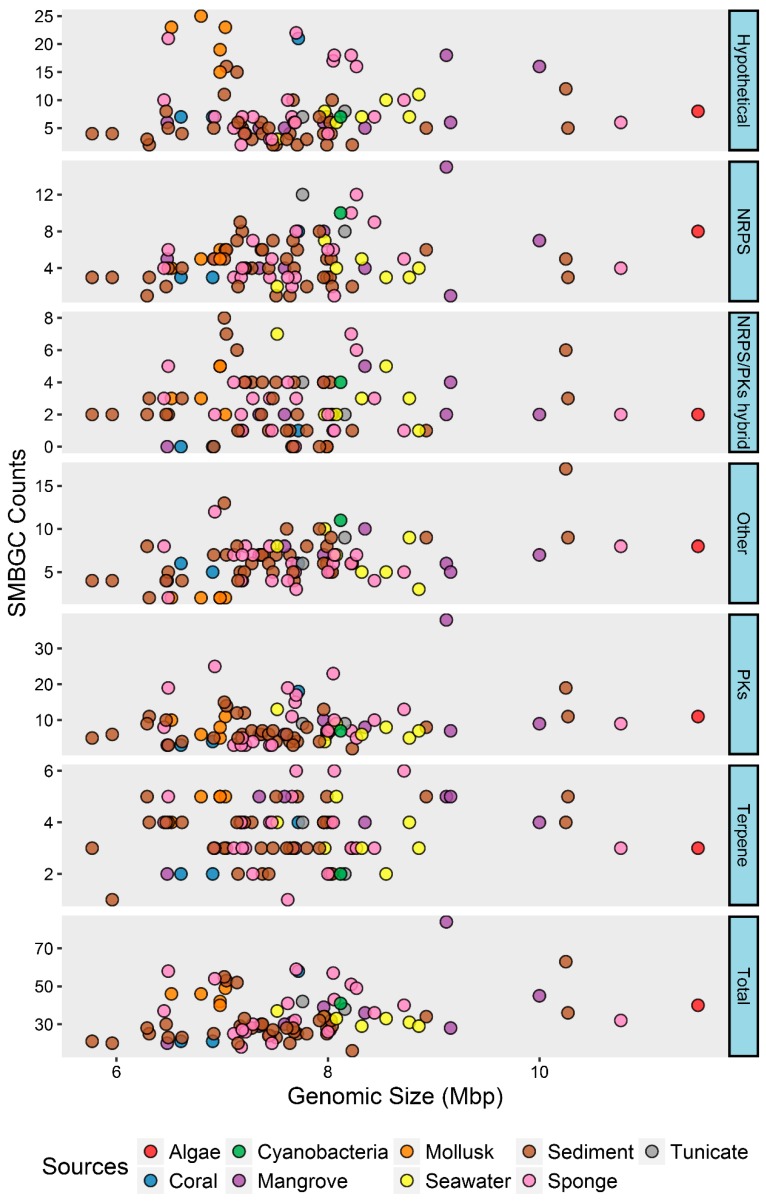
Secondary metabolism biosynthetic gene clusters (SMBGC) category counts identified in marine *Streptomyces* genomes.

It was detected that 16 to 84 SMBGCs (2 to 38 PKs, 1 to 15 NRPS, 0 to 8PKs/NRPS hybrid, 2 to 6 terpene, 2 to 17 other, and 2 to 25 hypothetical) were in the genomes of marine *Streptomyces* (Figure 2, Table S3) and the portion of SMBGCs in the genomes ranged from 1.94 to 9.21 Mbp^−1^, revealing that SMBGC counts were not positively correlated with genomic sizes (Figure 2), which is different from the correlation between gene counts and genomic size. Hence, SMBGCs distributions in the genomes of marine might be associated with their phylotypes and ecotypes, which could be intrinsic factors.

### 2.2. Comparative Genomics and Phylogenomic Relationship of Marine Streptomyces

**Figure 3:**
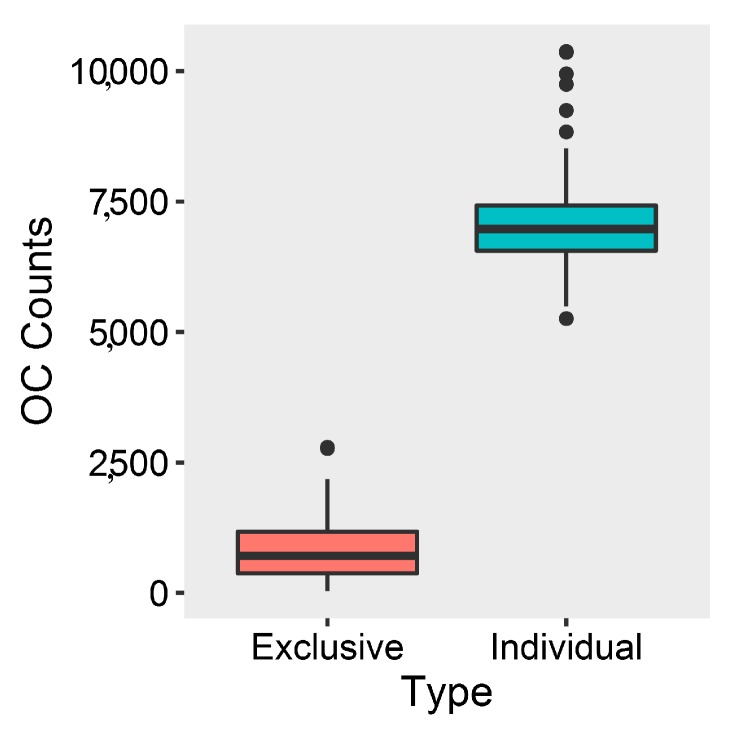
Individual and exclusive orthologous clusters (OCs) of marine *Streptomyces* genomes.

Comparative genomic analysis demonstrated that all of marine *Streptomyces* harbored 123,302 orthologous clusters (OCs) in their pan-genomes (Table S4), demonstrating their rich genetic diversities. Those strains contained 5258–10,376 OCs (average: 7116 ± 972, median: 6978) in their genomes, while they had 31–2793 (average: 861 ± 598, median: 714) exclusive OCs (Figure 3), also showing remarkable genetic diversities. It was detected that 996 OCs, of which 888 single-copy OCs were commonly in them and *Kitasatospora setae* KM-6054, were in their core-genomes (Table S4).

**Figure 4:**
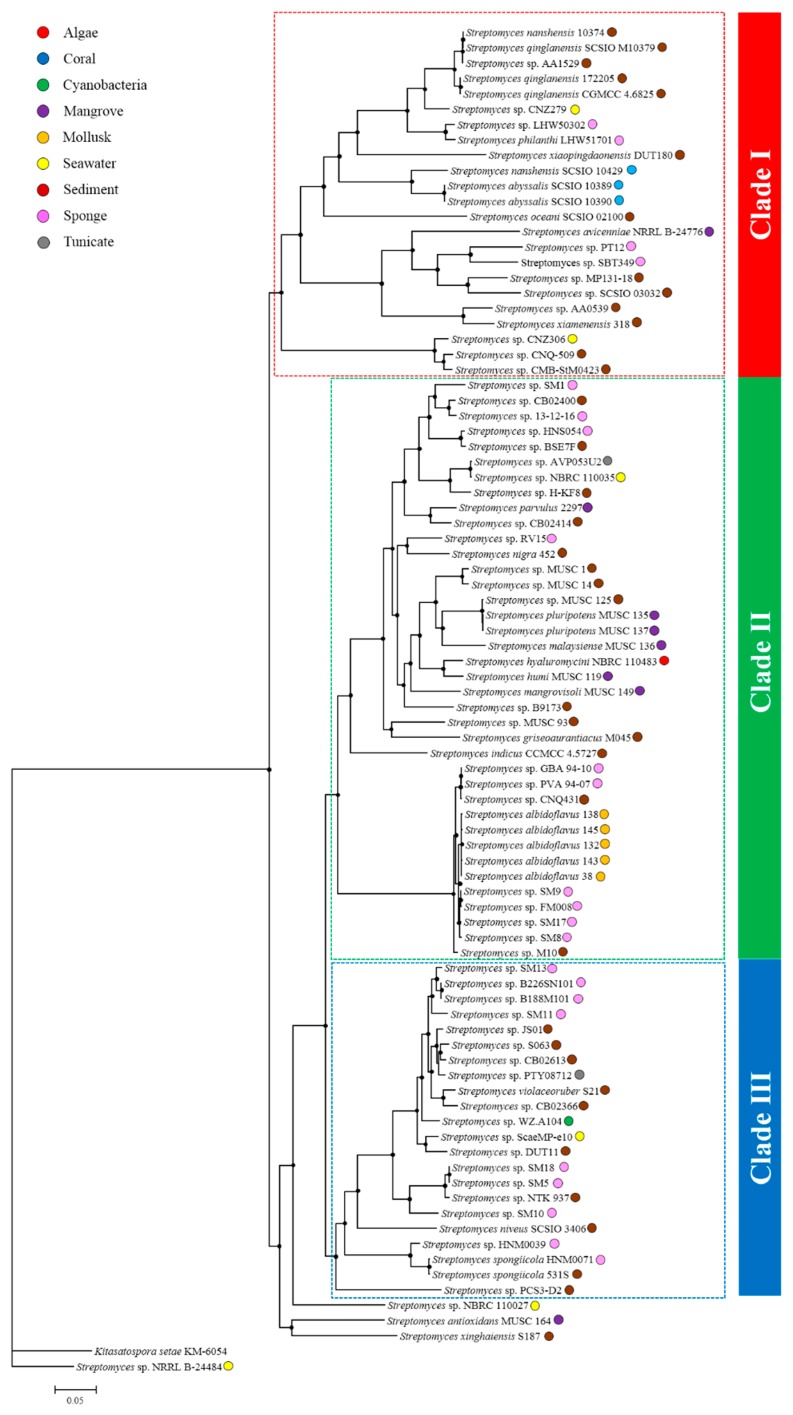
Maximum-likelihood phylogenomic tree based on the concatenation of 888 single-copy OC proteins shared by all of marine *Streptomyces*. Filled circle indicated nodes showing >85 of bootstrap values. *Kitasatospora setae* KM-6054 was used as an outgroup.

Based on the comparative genomic analysis, 888 single-copy OCs shared by all of marine *Streptomyces* and *Kitasatospora setae* KM-6054 (Table S4) were used to reconstruct a maximum-likelihood phylogenomic tree, revealing that the majority of marine *Streptomyces* were grouped into three clades (Clade I, II, and III) except for *S*. *antioxidans* MUSC 164, *S*. *xinghaiensis* S187, *Streptomyces* sp. NBRC 110027, and “*Streptomyces* sp. NRRL B-24484” (Figure 4). Further, “*Streptomyces* sp. NRRL B-24484” could not belong to the clade of the genus *Streptomyces*, which indicated that “*Streptomyces* sp. NRRL B-24484” was not a member of this genus, meaning it is excluded from further analysis.

Three major clades contain 23 (Clade I), 38 (Clade II), and 22 (Clade III) strains, respectively (Figure 4). Each clade consisted of strains derived from different sources, among which two majorities are marine sediment and sponge (Table 1). In addition, each clade had its own characteristic, which could be reflected by some ecotypes represented by ≥3 strains, such as coral- and mollusk-derived strains and only found in Clade I or II, and mangrove-derived strain only absent in the Clade III (Table 1).

Phylogenomic analysis also indicated that numerous novel *Streptomyces* species are waiting for identifications. Moreover, average nucleotide identity (ANI) calculations pointed out that Clade I, II, and III contained at least 9, 13 ,and 15 novel species (Table S5), which had low ANI values (<95%, [53]) compared with validly published *Streptomyces* species in the phylogenomic tree.

### 2.3. Phylotype-Associated SMBGCs

Except for hypothetical SMBGCs detected in the genomes of marine *Streptomyces*, significance tests among multi-clades revealed that 10 (6.0%) PKs, 7 (7.9%) NRPS, 3 (5.4%) PKs/NRPS hybrid, 2 (10.5%) terpene, and 7 (7.4%) other SMBGCs exhibited significant differences (Table 2). 

**Figure 5:**
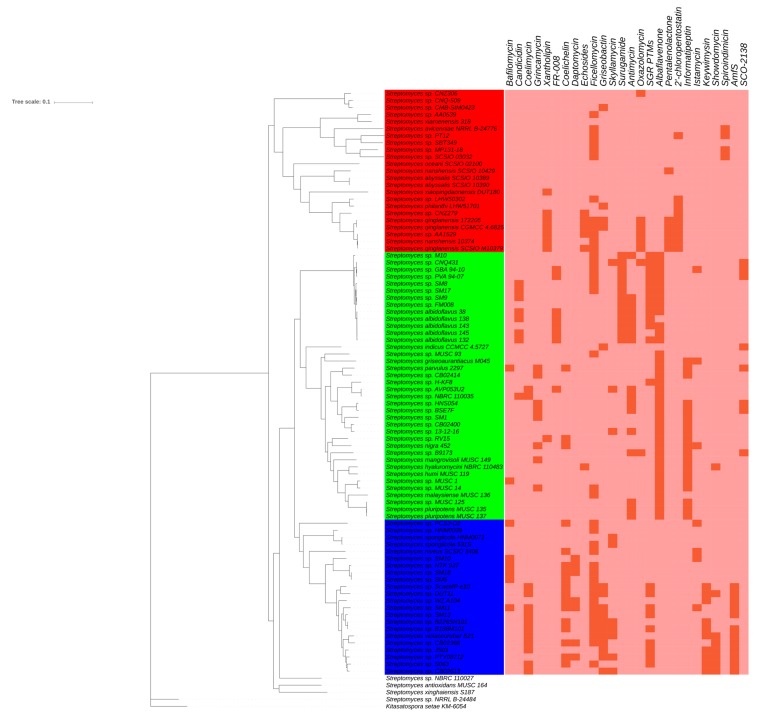
Heat maps of clade-specific SMBGCs. Dark brown and light brown indicate presence and absence of SMBGCs.

Among those SMBGCs, there were 23 SMBGCs showing clade-specificity (Figure 5), which is similar with previous studies regarding other genera [20,21]. (1) Marineosin, pentalenolactone, and spiroindimicin SMBGCs only appeared in the genomes of Clade I. (2) Albaflavenone, antimycin, candicidin, FR-008, grincamycin, informatipeptin, oxazolomycin, SCO-2138, as well as surugamide SMBGCs were exclusively present in the genomes of Clade II. (3) Amfs, daptomycin, and keywimysin SMBGCs was only found in the genomes of Clade III. (4) Bafilomycin, coelichelin, coelimycin, echosides, lactonamycin, SGR PTMs, skyllamycin, and xantholipin SMBGCs were present in the genomes of Clade II and III, while absent in the genomes of Clade I. It was observed that Clade I encoded more specific SMBGCs, whereas Clade II had more various SMBGCs than other two clades, which indicated that Clade II should be more valued. SMBGCs exclusively present in the genomes of Clade II could be classified into PKs, NRPS, PKs/NRPS hybrid, terpene, and others, showing the category diversities. Furthermore, products of those SMBGCs could exhibit antibacterial, antifungal, antitumor, and piscicide activities [58,60,66,75,76,78], highlighting their functional diversities.

### 2.4. Ecotype-Associated SMBGCs

Because strains that were derived from algae, corals, cyanobacteria, and tunicates are few, those strains were excluded from ecotype-associated analysis. Significance tests among multi-clades revealed that 8 (4.8%) PKs, 11 (12.4%) NRPS, 5 (9.0%) PKs/NRPS hybrid, 2 (10.5%) terpene, and 8 (8.4%) other SMBGCs exhibited significant differences (Table 3). Among those SMBGCs, albaflavenone, 2′-chloropentostatin, daptomycin, echosides, FR-008, oxazolomycin, and pentalenolactone SMBGCs showed significant differences among both of phylotypes and ecotypes.

**Figure 6:**
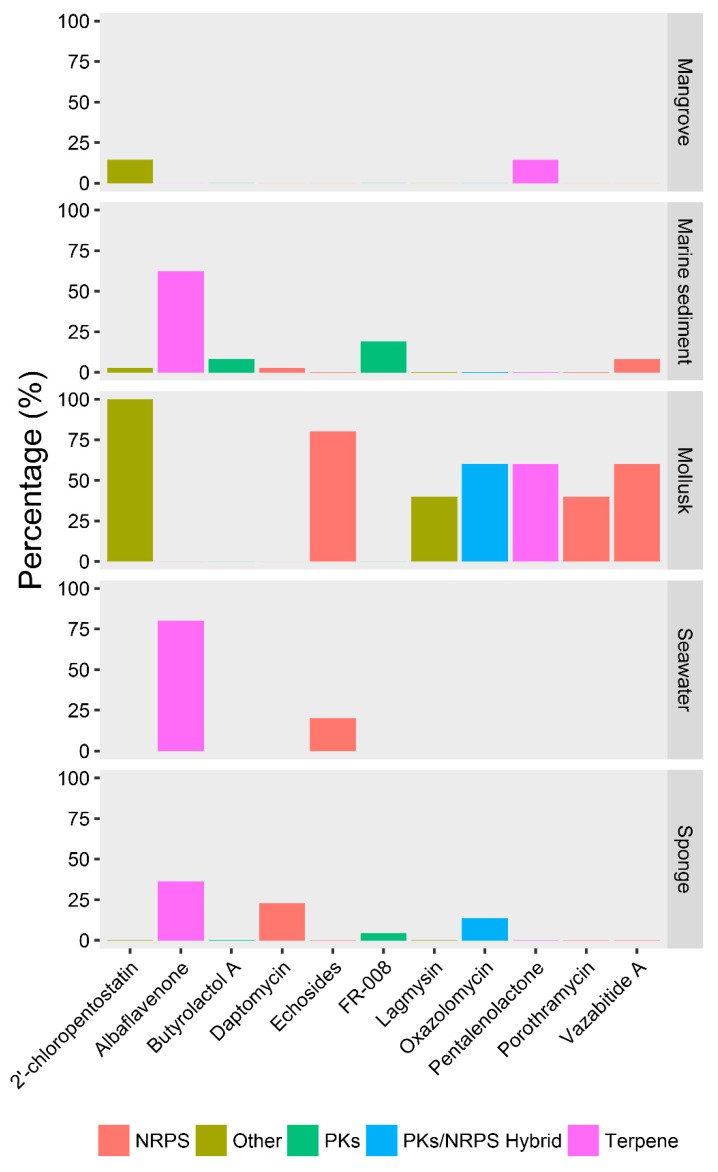
Distribution percentages of ecotype-specific SMBGCs.

Among those SMBGCs, there were 11 SMBGCs showing clade-specificity (Figure 6). (1) Butyrolactol and FR-008 SMBGCs commonly appeared in the genomes of marine sediment-derived strains; (2) Albaflavenone SMBGC were mostly found in the strains isolated from seawater and marine sediments. (3) Daptomycin SMBGC were associated with strains living in sponges. (4) 2′-chloropentostatin, echosides, lagmysin, oxazolomycin pentalenolactone, porothramycin, and vazabitide A SMBGCs were usually detected in the mollusk-derived strains. Marine sediment-derived strains were mostly related to several SMBGCs, making those strains appear to be specific in SMBGCs distribution patterns. Compared with natural environments, strains isolated from marine invertebrates, particularly for mollusks, had more SMBGCs, showing symbiotic *Streptomyces* in marine invertebrates could be profitable resources of secondary metabolites.

## 3. Materials and Methods

### 3.1. Obtain, Assess, and Annotate Marine Streptomyces Genomes

Ninety-seven available marine *Streptomyces* genomes were obtained from NCBI GenBank database in January, 2019 (Table S1). Genomic qualities of those genomes were assessed by using CheckM software v1.0.7 (Australian Centre for Ecogenomics, The University of Queensland, Queensland, Australia) [108] with the command “checkm lineage_wf -x fa bins/checkm/”, and those genomes exhibiting the completeness >95% as well as the contamination <5% were screened to the further study. 

rRNA genes were predicted by using with the command RNAmmer 1.2 package (Center for Biological Sequence Analysis, Technical University of Denmark, Lyngby, Denmark) “-S bac -m tsu,ssu,lsu” [109], while tRNA genes were annotated on the tRNAscan-SE On-line web server (http://trna.ucsc.edu/tRNAscan-SE/, [110]) with default mode except for the sequence source option set to “Bacterial”. Open reading frames (ORFs) were predicted and annotated on the RAST webserver (http://rast.nmpdr.org/rast.cgi, [111]). SMBGCs were annotated using antiSMASH bacterial version with detection strictness set to “relaxed” and extra features sletected to “ActiveSiteFinder, KnownClusterBlast, SubClusterBlast” (https://antismash.secondarymetabolites.org/#!/start, [112]). Moreover, functional annotations based on COG and KEGG databases were carried out on WebMGA (http://weizhong-lab.ucsd.edu/webMGA/server/cog/) and KASS (https://www.genome.jp/tools/kaas/) webservers [113,114]. The GC content of those marine *Streptomyces* genomes were calculated by using OrthoANI [115].

### 3.2. Comparative Genomic Analysis of Marine Streptomyces Genomes

*Kitasatospora setae* KM-6054^T^ was used as an outgroup in the further phylogenomic analysis based on recent polyphasic taxonomic studies [116,117,118], so its genome, which is under the NCBI GenBank assembly accession number of GCA_000269985.1, was also included in comparative genomic analysis. Comparative genomic analysis was modified based on the method described by Xu et al. [119]. Protein sequences translated from ORFs were compared pairwise using Proteinortho V5.16b (Interdisciplinary Center for Bioinformatics, University of Leipzig, Leipzig, Germany) with the command “-cov = 50 -identity = 50” [120] to identify OCs among genomes of marine *Streptomyces* and their outgroup. A set of OCs are defined as a class of genes transferred vertically from a common descent [121]. 

### 3.3. Phylogenomic Analysis and Genomic Similarity Calculation of Marine Streptomyces

Single-copy OCs shared by all of marine *Streptomyces* as well as *Kitasatospora setae* KM-6054^T^ were screened by in-house perl script. Each single-copy OCs was aligned by using MAFFT version 7 (Computational Biology Research Center, The National Institute of Advanced Industrial Science and Technology, Tokyo, Japan) with the command “--auto” [122]. Then, aligned sequences were refined to remove poorly aligned regions by trimAL version 1.4.1 with the command “-automated1” [123], and concatenated manually. Subsequently, a maximum-likelihood phylogenomic tree based on concatenated protein sequences was reconstructed by using IQ-Tree 1.6.1 software (Center for Integrative Bioinformatics Vienna, Max F. Perutz Laboratories, University of Vienna, Medical University of Vienna, Vienna, Austria) with ultrafast bootstraps analysis set to 1000 replicates [124,125,126], following the best amino acid substitution model set as LG+F+R8 proposed by IQ-Tree 1.6.1 software with the command “-st AA -m MFP” [125]. 

Genome similarities of pairwise marine *Streptomyces* genomes were determined by calculating ANI values, which were obtained by using orthologous average nucleotide identity tool (OAT) 0.93.1 (Chunlab Inc., Seoul, Korea) [115] supplemented with basic local alignment search tool (BLAST) algorithm [127].

### 3.4. Statistical Analysis and Visualization

Unless stated, statistical analyses were performed by using R version 3.4.2 (R Foundation for Statistical Computing) [128]. Correlation of genomic size and gene counts were analyzed by using the function of *lm*. Significance test analyses of SMBGCs among phylotypes and ecotypes were performed by using the function of *kruskal.test*, with *p* values <0.01 showing the significant difference. Pan- and core-genomic analysis were carried out by summarizing OCs counts by using “*grep*” command in the CentOS 6 system (Red Hat, Inc., Raleigh, NC, USA).

The phylogenomic tree was visualized by using MEGA 7 software [129] and PowerPoint 2016 software (Microsoft Cooperation, Redmond, WA, USA). Unless heat maps were drawn by using Interactive Tree Of Life webserver (https://itol.embl.de/), other figures were constructed by using *ggplot2* and *Cairo* packages in R version 3.4.2 [128].

## 4. Conclusions

Marine *Streptomyces* is characterized by its rich species, genetic, and secondary metabolism diversities. Comparative genomics of Marine *Streptomyces* revealed that those group have a wide range of OCs showing high genetic diversity. Phylogenomic analysis in this study shows that enormous novel marine *Streptomyces* species needs to be identified and the majority can be classified into three clades. Phylotype and ecotype are both associated with SMBGCs distribution patterns. The Clade I and marine sediment-derived *Streptomyces* harbored more specific SMBGCs, which consisted of several common ones, such as butyrolactol, FR-008, marineosin, pentalenolactone, and spiroindimicin, whereas the Clade II and marine invertebrate-derived *Streptomyces* have more SMBGCs, such as 2′-chloropentostatin, albaflavenone, antimycin, candicidin, echosides, FR-008, grincamycin, informatipeptin, lagmysin, oxazolomycin, pentalenolactone, porothramycin, SCO-2138, and vazabitide A, indicating that those *Streptomyces* could act as plentiful resources for mining secondary metabolites. As stated above, our study is beneficial for broadening our knowledge about SMBGC distribution patterns in marine *Streptomyces* and developing their secondary metabolites in the future.

## Figures and Tables

**Table 1:** The percentage of sources in Clade I, II, and II.

Clade	1	2	3	4	5	6	7	8	9
I	0%	13%	0%	4%	0%	9%	56%	18%	0%
II	3%	0%	0%	16%	13%	5%	37%	23%	3%
III	0%	0%	4%	0%	0%	4%	46%	42%	4%

1, 2, 3, 4, 5, 6, 7, 8, and 9 represents algae, coral, cyanobacteria, mangrove, mollusk, seawater, marine sediment, sponge, and tunicate, respectively.

**Table 2:** SMBGCs showing significant differences among three major clades of marine *Streptomyces*.

SMBGC		*p* Value	Activity
PKs			
	Alkylresorcinol	0.003	Prevention of tumor [54]
	Bafilomycin	0.003	Antiprozoan [55] Antitumor [56] Immunosuppressant [57]
	Candicidin	0.005	Antifungus [58]
	Coelimycin	1.60 × 10^−6^	Yellow pigment [59]
	Grincamycin	0.005	Antitumor [60]
	Lactonamycin	0.005	Antibacteria [61]
	Marineosin	0.004	Cytotoxicity [62]
	Steffimycin	2.43 × 10^−7^	Antitumor [63]
	Spore pigment	0.0002	Regulate sporulation [64]
	Xantholipin	0.0003	Antibacteria [65] Cytotoxicity [65]
	FR-008	0.005	Antifungus [66]
NRPS			
	Coelichelin	3.22 × 10^−8^	Siderophore [67]
	Daptomycin	2.84 × 10^−5^	Antibacteria [68]
	Echosides	0.006	Antivirus [69]
	Ficellomycin	0.003	Antibacteria [70]
	Griseobactin	4.72 × 10^−5^	Siderophore [71]
	Skyllamycin	0.003	Antitumor [72] Antibacteria [73]
	Surugamide	0.0001	Antibacteria [74]
PKs/NRPS hybrid			
	Antimycin	4.24 × 10^−6^	Piscicide [75]
	Oxazolomycin	0.005	Antibacteria [76]
	SGR PTMs	0.003	Antifungal [77] Antioxidant [77]
Terpene			
	Albaflavenone	2.20 × 10^−16^	Antibacteria [78]
	Pentalenolactone	0.002	Immunosuppressant [79]
Other			
	2′-chloropentostatin	2.2 × 10^−16^	Antivirus [80]
	Informatipeptin	5.50 × 10^−7^	Unknown
	Keywimysin	1.72 × 10^−7^	Unknown
	Melanin	4.58 × 10^−6^	Antioxidant [81]
	Showdomycin	0.0002	Antitumor [82]
	Spiroindimicin	0.004	Cytotoxicity [83]
	AmfS	4.52 × 10^−9^	Morphogen [84]
	SCO-2138	0.006	Unknown

**Table 3:** SMBGCs showing significant differences among ecotypes of marine *Streptomyces*.

SMBGC		*p* Value	Activity
PKs			
	Brasilinolide	0.007	Antifungus [85]
	Butyrolactol A	0.0008	Antifungus [86]
	Incednine	0.007	Antiapotosis [87]
	Micromonolactam	0.0009	Unknown
	Napyradiomycin	0.007	Antibacteria [88] Cytotoxicity [88]
	Rifamycin	0.007	Antibacteria [89]
	Vicenistatin	0.007	Antitumor [90]
	FR-008	5.14 × 10^−11^	Antifungus [66]
NRPS			
	Actinomycin	0.009	Antibacteria [91] Antitumor [91]
	Daptomycin	0.007	Antibacteria [68]
	Echosides	4.07 × 10^−8^	Antivirus [69]
	Porothramycin	6.81 × 10^−5^	Antibacteria [92] Antitumor [92]
	Rhizomide	0.008	Antibacteria [93]
	Scabichelin	0.009	Siderophore [94]
	Stenothricin	0.003	Antibacteria [95]
	Syringomycin	0.0005	Antifungus [96]
	Telomycin	0.0005	Antibacteria [97]
	Vazabitide A	0.002	Immunosuppressant [98]
	Yatakemycin	1.21 × 10^−6^	Cytotoxicity [99]
PKs/NRPS hybrid			
	Ansatrienin	7.44 × 10^−11^	Antibacteria [100]
	Kistamicin A	0.003	Antibacteira [101]
	Oxazolomycin	6.60 × 10^−5^	Antibacteria [76]
	Rakicidin	1.07 × 10^−5^	Antitumor [102]
	Zorbamycin	1.21 × 10^−6^	Antitumor [103]
Terpene			
	Albaflavenone	0.003	Antibacteria [78]
	Pentalenolactone	8.94 × 10^−7^	Immunosuppressant [79]
Other			
	2′-chloropentostatin	1.50 × 10^−9^	Antivirus [80]
	Clavulanic acid	0.003	Antibacteria [104]
	Desferrioxamine	0.009	Antitumor [105]
	Lagmysin	6.81 × 10^−5^	Unknown
	Legonaridin	1.21 × 10^−6^	Cytotoxicity [106]
	Marinophenazines	0.005	Unknown
	Roseoflavin	0.0002	Antibacteria [107]

## Supplementary Materials

The following are available online at https://www.mdpi.com/1660-3397/17/9/498/s1, Table S1. Genomic information and quality estimations of marine Streptomyces obtained from NCBI GenBank database. Table S2. Genomic annotations of marine *Streptomyces* by using RAST webserver. Table S3. SMBGC annotations of marine *Streptomyces* by using antiSMASH webserver. Table S4. OCs distributions in marine *Streptomyces*. Table S5. ANI values among marine *Streptomyces*.
