# Supplementary material for: Comparative Genomic Insights into Secondary Metabolism Biosynthetic Gene Cluster Distributions of Marine Streptomyces
Source: Mar Drugs. 2019 Aug 26;17(9):498. doi: 10.3390/md17090498 (PMC6780079; doi:10.3390/md17090498)
Supplement: Supplementary file 1 [file marinedrugs-17-00498-s001.zip › supplementary/Comparative_Genomics_Marine_Streptpmyces-SM-Table_S1 and Table_S2.pdf]

Table 1. Genomic information and quality estimations of marine *Streptomyces* obtained from NCBI GenBank database.

**Strain**	**NCBI GenBank assembly accession number**	**Isolation environment**	**Contigs**	**Size (Mbp)**	**GC content (mol%)**	**Completeness (%)**	**Contamination (%)**
Streptomyces abyssalis SCSIO 10389	GCA_001751345.1	Marine coral	45	6.91	70.3	99.2	0.4
Streptomyces abyssalis SCSIO 10390	GCA_001751365.1	Marine coral	42	6.61	70.9	99.5	0
Streptomyces_albidoflavus 38	GCA_002317005.1	Sea snail	1114	6.98	73.0	99.7	1.0
Streptomyces albidoflavus 132	GCA_002289335.1	Sea snail	1053	6.80	73.0	98.7	1.2
Streptomyces albidoflavus 138	GCA_002317025.1	Sea snail	1028	6.98	73.0	98.8	0.4
Streptomyces albidoflavus 143	GCA_002317035.1	Sea snail	957	7.03	73.0	99.2	0.2
Streptomyces albidoflavus 145	GCA_002289305.1	Sea snail	1153	6.52	73.0	98.4	0.4
Streptomyces antioxidans MUSC 164	GCA_000968685.2	Mangrove	282	9.12	71.5	100.0	4.2
Streptomyces avicenniae NRRL B-24776	GCA_000719135.1	Mangrove	66	6.48	73.5	95.5	0.9
Streptomyces chilikensis RC 1830	GCA_001642695.1	Sediment	1484	4.87	69.8	67.8	1.9
Streptomyces diastaticus deep-sea A18	GCA_002892485.1	Sediment	1377	7.71	71.7	100	8.6
Streptomyces gilvigriseus MUSC 26	GCA_001879105.1	Mangrove	206	5.21	73.0	94.7	0
Streptomyces griseoaurantiacus M045	GCA_000204605.2	Sediment	46	7.71	72.7	100.0	0
Streptomyces humi MUSC 119	GCA_001005085.2	Mangrove	214	10.0	71.8	100.0	0.2
Streptomyces hyaluromycini NBRC 110483	GCA_002217755.1	Sea grapes	52	11.5	71.1	100.0	0.1
Streptomyces indicus CGMCC 4.5727	GCA_900100315.1	Sediment	45	8.23	71.4	99.9	1.0
Streptomyces malaysiense MUSC 136	GCA_000980885.2	Mangrove	235	7.96	72.2	99.6	0.3
Streptomyces mangrovisoli MUSC 149	GCA_000974985.2	Mangrove	199	9.16	72.5	100.0	1.1
Streptomyces nanshensis 10374	GCA_001753705.1	Sediment	67	6.49	72.4	98.8	0
Streptomyces nanshensis SCSIO 01066	GCA_001751275.1	Sediment	2310	7.43	70.3	100.0	9.3
Streptomyces nanshensis SCSIO 10429	GCA_001751255.1	Marine coral	731	7.72	71.6	99.0	1.7
Streptomyces nanshensis SCSIO M10372	GCA_001751305.1	Sediment	114	10.27	70.9	98.0	6.9
Streptomyces nanshensis SCSIO M10399	GCA_001751265.1	Sediment	1285	8.58	58.4	91.4	9.0
Streptomyces nigra 452	GCA_003074055.1	Sediment	1	7.64	71.9	99.7	0.2
Streptomyces niveus SCSIO 3406	GCA_002009175.1	Sediment	1	7.99	70.5	99.5	0.7
Streptomyces oceani SCSIO 02100	GCA_001751245.1	Sediment	166	6.31	70.4	98.1	0.9
Streptomyces parvulus 2297	GCA_003344905.1	Mangrove	118	8.35	72.3	100	1.4
Streptomyces philanthi LHW51701	GCA_003323715.1	Marine sponge	80	7.66	72.2	99.6	1.4
Streptomyces pluripotens MUSC 135	GCA_000802245.2	Mangrove	1	7.35	69.9	99.5	0.6
Streptomyces pluripotens MUSC 137	GCA_000816465.4	Mangrove	1	7.59	69.9	99.5	0.6
Streptomyces qinglanensis 172205	GCA_003265665.1	Sediment	38	7.19	72.6	98.9	1.0
Streptomyces qinglanensis CGMCC 4.6825	GCA_900111245.1	Sediment	34	7.17	72.7	99.3	1.0
Streptomyces qinglanensis SCSIO M10379	GCA_001751375.1	Sediment	22	6.61	72.8	98.8	0.3

Streptomyces sp. 13-12-16	GCA_002114215.1	Marine sponge	1513	8.72	71.6	96.5	1.2
Streptomyces sp. AA0539	GCA_000297635.1	Sediment	57	5.77	72.9	95.8	0.9
Streptomyces sp. AA1529	GCA_000280905.1	Sediment	109	7.28	72.7	98.8	0.2
Streptomyces sp. AVP053U2	GCA_001469455.2	Sea Squirt	183	7.76	71.9	100.0	1.2
Streptomyces sp. B188M101	GCA_002910985.1	Marine sponge	609	8.22	71.6	99.5	0.2
Streptomyces sp. B226SN101	GCA_002910935.1	Marine sponge	580	8.27	71.6	99.8	0.2
Streptomyces sp. B9173	GCA_002081495.1	Seawater	133	8.77	71.8	100.0	0
Streptomyces sp. BSE7F	GCA_003122045.1	Sediment	5	7.51	72.3	98.1	0.4
Streptomyces sp. CB02366	GCA_001905905.1	Beach sand	44	7.37	72.5	99.9	0.3
Streptomyces sp. CB02400	GCA_001905725.1	Beach sand	47	8.93	71.7	100.0	0
Streptomyces sp. CB02414	GCA_001905385.1	Beach sand	19	7.99	72.0	100.0	0.6
Streptomyces sp. CB02613	GCA_002803175.1	Beach sand	28	7.38	71.7	99.5	0
Streptomyces sp. CMB-StM0423	GCA_002847285.1	Beach sand	1	8.03	73.1	100	1.3
Streptomyces sp. CNQ431	GCA_000797385.1	Sediment	295	7.04	73.1	99.9	0.1
Streptomyces sp. CNQ-509	GCA_001011035.1	Sediment	1	8.04	73.1	100.0	1.6
Streptomyces sp. CNZ279	GCA_002754675.1	Seawater	2	7.97	71.1	97.0	1.4
Streptomyces sp. CNZ306	GCA_002797655.1	Seawater	5	8.55	73.5	100	0.5
Streptomyces sp. DUT11	GCA_002848525.1	Sediment	1	8.03	71.8	97.3	1.0
Streptomyces sp. FM008	GCA_002910815.1	Marine sponge	1369	6.48	72.7	96.4	0.8
Streptomyces sp. GBA 94-10	GCA_000495635.1	Marine sponge	2	7.22	73.0	99.9	0.1
Streptomyces sp. H-KF8	GCA_001672315.1	Sediment	11	7.68	72.1	100.0	0.5
Streptomyces sp. HNM0039	GCA_003097515.1	Marine sponge	1	7.29	72.5	99.5	0.4
Streptomyces sp. HNS054	GCA_001044185.1	Marine sponge	117	7.45	72.4	99.6	0
Streptomyces sp. JS01	GCA_000743295.1	Sediment	38	7.80	71.6	99.8	0.4
Streptomyces sp. LHW50302	GCA_003323735.1	Marine sponge	70	7.69	72.0	99.6	1.9
Streptomyces sp. M10	GCA_000800535.1	Sediment	77	7.21	73.3	99.4	0
Streptomyces sp. MP131-18	GCA_001984575.1	Sediment	10	7.96	72.0	97.4	1.6
Streptomyces sp. MUSC 1	GCA_001866665.1	Sediment	218	10.25	71.5	99.9	1.4
Streptomyces sp. MUSC 14	GCA_001866675.1	Sediment	174	10.27	71.3	100.0	1.4
Streptomyces sp. MUSC 93	GCA_001866645.1	Sediment	166	7.02	69.9	99.4	1.8
Streptomyces sp. MUSC 125	GCA_000816485.1	Sediment	164	7.66	70.0	99.5	0.4
Streptomyces sp. NBRC 110027	GCA_000829715.2	Seawater	20	8.32	71.1	100.0	1.5
Streptomyces sp. NBRC 110035	GCA_000829695.1	Seawater	89	7.51	71.8	100.0	0.8
Streptomyces sp. NRRL B-24484	GCA_000717715.1	Seawater	186	8.86	73.8	99.5	1.8
Streptomyces sp. NRRL B-24501	GCA_000716085.1	Seawater	448	11.56	68.0	83.7	0.5
Streptomyces sp. NTK 937	GCA_000698495.1	Sediment	3	7.44	71.9	99.8	0.1
Streptomyces sp. PCS3-D2	GCA_000612545.1	Sediment	27	7.48	72.0	99.9	0.5
Streptomyces sp. PT12	GCA_003311645.1	Marine sponge	296	6.92	72.9	96.1	1.6

Streptomyces sp. PTY087I2	GCA_001687325.1	Sea squirt	83	8.16	71.5	99.8	0.5
Streptomyces sp. PVA_94-07	GCA_000495755.1	Marine sponge	3	7.11	73.1	99.9	0.1
Streptomyces sp. RV15	GCA_001514305.1	Marine sponge	152	10.77	70.2	100	1.2
Streptomyces sp. S063	GCA_002832675.1	Sediment	1	7.61	71.5	99.0	0
Streptomyces sp. SBT349	GCA_001083795.1	Marine sponge	691	8.06	72.7	95.1	1.1
Streptomyces sp. ScaeMP-e10	GCA_000373405.1	Seawater	20	8.08	71.6	99.8	1.0
Streptomyces sp. SCSIO_03032	GCA_002128305.1	Sediment	1	6.29	73.5	96.5	1.4
Streptomyces sp. SHP22-7	GCA_003573595.1	Sediment	146	7.90	72.2	89.0	0.5
Streptomyces sp. SM1	GCA_002910825.1	Marine sponge	1057	8.06	71.1	99.9	0.3
Streptomyces sp. SM5	GCA_002910895.1	Marine sponge	496	7.61	71.7	99.8	0.8
Streptomyces sp. SM8	GCA_000299175.2	Sediment	11	7.14	73.2	96.2	0.4
Streptomyces sp. SM9	GCA_002910795.1	Marine sponge	1592	6.45	72.7	97.0	0.8
Streptomyces sp. SM10	GCA_002910915.1	Marine sponge	195	7.47	70.8	99.5	0.4
Streptomyces sp. SM11	GCA_002910905.1	Marine sponge	311	8.00	71.1	99.3	1.1
Streptomyces sp. SM12	GCA_002910855.1	Marine sponge	910	6.48	72.2	94.3	0.6
Streptomyces sp. SM13	GCA_002910875.1	Marine sponge	388	8.44	71.2	99.8	0.2
Streptomyces sp. SM14	GCA_002910755.1	Marine sponge	639	6.34	72.4	94.8	1.1
Streptomyces sp. SM17	GCA_002910725.2	Marine sponge	4	7.18	73.3	99.9	0.4
Streptomyces sp. SM18	GCA_002910775.2	Marine sponge	1	7.70	71.8	99.8	0.8
Streptomyces sp. WZ.A104	GCA_002382885.1	Marine cyanobacteria	189	8.12	71.2	99.7	1.5
Streptomyces spongiicola 531S	GCA_003402595.1	Sediment	84	6.91	72.6	99.4	0
Streptomyces spongiicola HNM0071	GCA_003122365.1	Marine sponge	1	7.18	72.4	99.4	0.2
Streptomyces violaceoruber S21	GCA_002082175.1	Sediment	1	7.92	72.7	99.9	0.5
Streptomyces xiamenensis 318	GCA_000993785.2	Sediment	1	5.96	72.0	95.8	0.5
Streptomyces xiaopingdaonensis DUT180	GCA_000262345.1	Sediment	385	6.47	72.2	99.5	0.6
Streptomyces xinghaiensis S187	GCA_000220705.2	Sediment	1	7.14	73.1	100.0	1.3

Table S2. Genomic annotations of marine *Streptomyces* by using RAST webserver.

**Strain**	**ORFs**	**rRNA Genes**	**tRNA Genes**
Streptomyces abyssalis SCSIO 10389	6185	17	57
Streptomyces abyssalis SCSIO 10390	5961	13	54
Streptomyces albidoflavus 38	7006	8	66
Streptomyces albidoflavus 132	6873	8	69
Streptomyces albidoflavus 138	6973	8	70
Streptomyces albidoflavus 143	6988	8	69

Streptomyces albidoflavus 145	6486	1	62
Streptomyces antioxidans MUSC 164	7845	6	61
Streptomyces avicenniae NRRL B-24776	6234	2	53
Streptomyces griseoaurantiacus M045	7108	5	66
Streptomyces humi MUSC 119	9020	4	68
Streptomyces hyaluromycini NBRC 110483	10681	4	72
Streptomyces indicus CGMCC 4.5727	7667	6	63
Streptomyces malaysiense MUSC 136	7529	3	66
Streptomyces mangrovisoli MUSC 149	8266	6	68
Streptomyces nanshensis 10374	5697	16	60
Streptomyces nanshensis SCSIO 10429	7422	9	59
Streptomyces nigra 452	7114	18	68
Streptomyces niveus SCSIO 3406	7285	18	64
Streptomyces oceani SCSIO 02100	5650	3	56
Streptomyces parvulus 2297	7914	5	63
Streptomyces philanthi LHW51701	6875	8	58
Streptomyces pluripotens MUSC 135	6813	18	67
Streptomyces pluripotens MUSC 137	7068	18	67
Streptomyces qinglanensis 172205	6605	3	58
Streptomyces qinglanensis CGMCC 4.6825	6593	8	59
Streptomyces qinglanensis SCSIO M10379	5783	15	58
Streptomyces sp. 13-12-16	9414	6	68
Streptomyces sp. AA0539	5305	3	55
Streptomyces sp. AA1529	6716	5	58
Streptomyces sp. AVP053U2	7334	9	68
Streptomyces sp. B188M101	7958	20	66
Streptomyces sp. B226SN101	7900	20	65
Streptomyces sp. B9173	8092	3	72
Streptomyces sp. BSE7F	7294	21	66
Streptomyces sp. CB02366	6910	4	65
Streptomyces sp. CB02400	8375	3	69
Streptomyces sp. CB02414	7441	4	66
Streptomyces sp. CB02613	6738	4	64
Streptomyces sp. CMB-StM0423	7080	15	55
Streptomyces sp. CNQ431	6603	3	47
Streptomyces sp. CNQ-509	7223	15	56
Streptomyces sp. CNZ279	7535	14	58
Streptomyces sp. CNZ306	7461	17	58

Streptomyces sp. DUT11	8354	18	64
Streptomyces sp. FM008	6863	14	65
Streptomyces sp. GBA 94-10	6529	21	65
Streptomyces sp. H-KF8	7182	18	66
Streptomyces sp. HNM0039	6673	16	67
Streptomyces sp. HNS054	7094	3	67
Streptomyces sp. JS01	7291	4	71
Streptomyces sp. LHW50302	7119	3	64
Streptomyces sp. M10	6,514	2	54
Streptomyces sp. MP131-18	7,360	2	54
Streptomyces sp. MUSC 1	9,994	4	66
Streptomyces sp. MUSC 14	10,218	4	67
Streptomyces sp. MUSC 93	6,851	5	66
Streptomyces sp. MUSC 125	7,328	4	66
Streptomyces sp. NBRC 110027	7,616	9	66
Streptomyces sp. NBRC 110035	7,113	14	65
Streptomyces sp. NRRL B-24484	8,690	6	68
Streptomyces sp. NTK 937	6,804	18	81
Streptomyces sp. PCS3-D2	6,973	3	69
Streptomyces sp. PT12	6,368	2	53
Streptomyces sp. PTY087I2	7,398	9	65
Streptomyces sp. PVA_94-07	6,334	21	65
Streptomyces sp. RV15	10,708	3	65
Streptomyces sp. S063	7,330	18	65
Streptomyces sp. SBT349	7,627	2	54
Streptomyces sp. ScaeMP-e10	7,356	7	66
Streptomyces sp. SCSIO_03032	5,654	10	54
Streptomyces sp. SM1	8,483	19	68
Streptomyces sp. SM5	7,325	16	65
Streptomyces sp. SM8	6,783	4	60
Streptomyces sp. SM9	6,931	18	63
Streptomyces sp. SM10	7,059	30	67
Streptomyces sp. SM11	7,838	13	68
Streptomyces sp. SM13	8,329	16	66
Streptomyces sp. SM17	6,517	21	66
Streptomyces sp. SM18	7,025	18	67
Streptomyces sp. WZ.A104	7,708	4	66
Streptomyces spongiicola 531S	6,416	5	63

Streptomyces spongiicola HNM0071	6,577	18	65
Streptomyces violaceoruber S21	7,179	18	65
Streptomyces xiamenensis 318	5,547	15	55
Streptomyces xiaopingdaonensis DUT180	6,267	2	47
Streptomyces xinghaiensis S187	6,503	18	63
